# Supplementary material for: Efficacy and Safety of a Single Ivy Extract Versus Two Herbal Extract Combinations in Patients with Acute Bronchitis: A Multi-Center, Randomized, Open-Label Clinical Trial
Source: Pharmaceuticals (Basel). 2025 May 20;18(5):754. doi: 10.3390/ph18050754 (PMC12114782; doi:10.3390/ph18050754)
Supplement: Supplementary file 1 [file pharmaceuticals-18-00754-s001.zip › pharmaceuticals-3636837-supplementary.pdf]

a

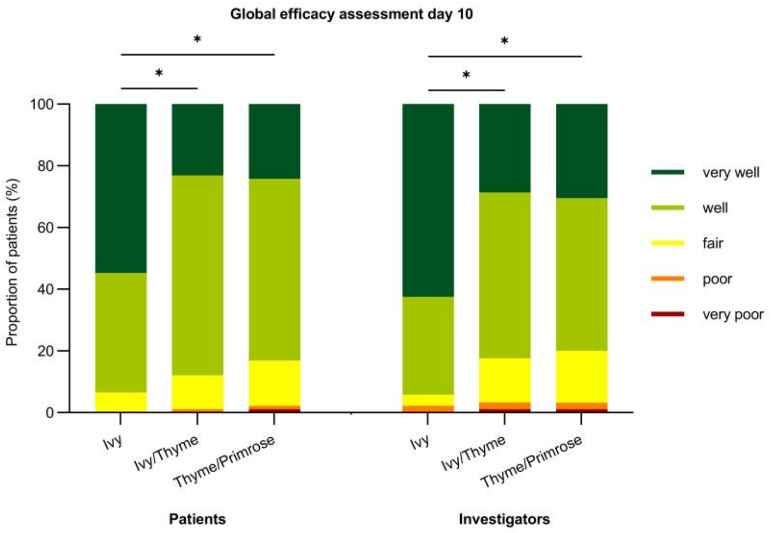

b

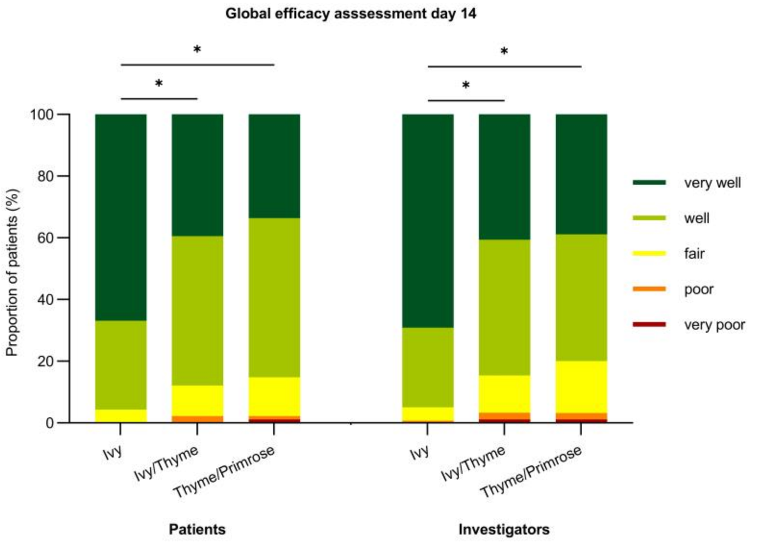

Supplementary Figure S1. Assessment of global efficacy by patients (Considering all the ways this treatment has affected you since you started in the trial, how well are you doing?) and investigators (How do you rate this medication as a treatment for bronchitis?) on day 10 (a) and day 14 (b) for the modified full analysis set (mFAS) (*p < 0.0001).

a

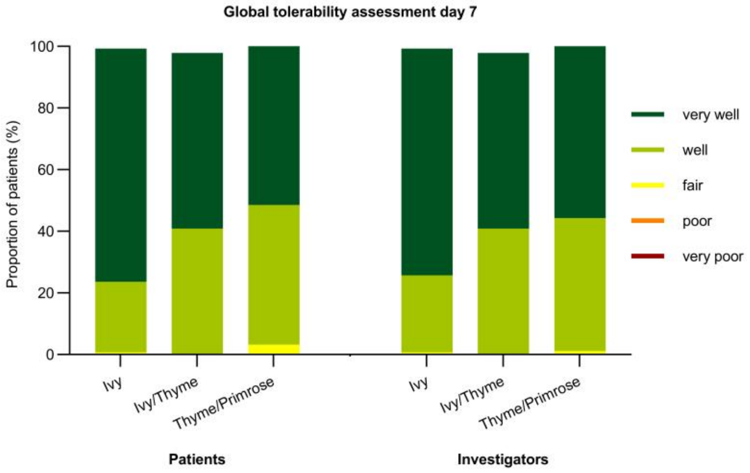

b

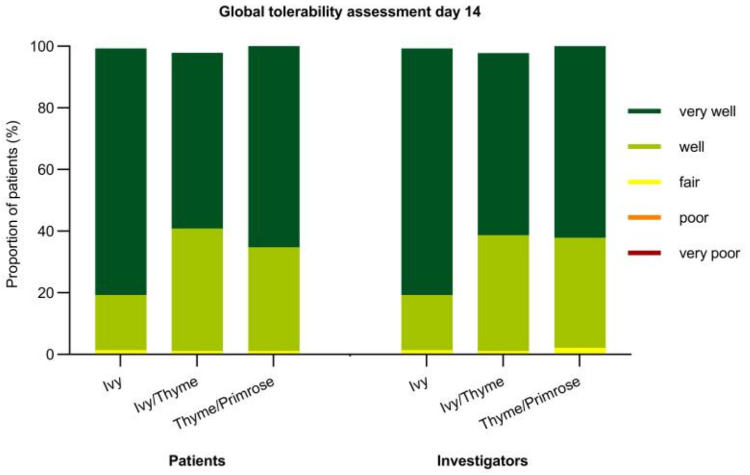

Supplementary Figure S2. Assessment of global tolerability by patients (Considering all the ways this treatment has affected you since you started in the trial, how well did you tolerate the treatment?) and investigators (Considering all the ways this treatment has affected the patient since he/she started in the trial, how well did he/she tolerate the treatment?) on day 7 (a) and day 14 (b) for the modified full analysis set (mFAS).
